# Total Cerebral Small Vessel Disease Burden on MRI Correlates With Cognitive Impairment in Outpatients With Amnestic Disorders

**DOI:** 10.3389/fneur.2021.747115

**Published:** 2021-12-02

**Authors:** Yangyi Fan, Yicheng Xu, Ming Shen, Huailian Guo, Zhaoxu Zhang

**Affiliations:** ^1^Department of Neurology, Peking University People's Hospital, Beijing, China; ^2^Department of Neurology, Aerospace Center Hospital, Beijing, China

**Keywords:** cerebral small vessel disease, brain magnetic resonance imaging, cognitive impairment, white matter hyperintensities, enlarged perivascular spaces, cerebral microbleed, lacune

## Abstract

**Objectives:** The main markers of cerebral small vessel disease (cSVD) on MRI may be entered into a scoring system, with the total score representing the overall burden of cSVD. An association between total cSVD score and cognitive dysfunction has been reported in several cohorts. The present study aimed to investigate this association in outpatients with amnestic disorders.

**Materials and Methods:** Outpatients with amnestic complaints in a memory clinic (*n* = 289) were recruited retrospectively. All the patients had undergone clinical and cognitive evaluation at first presentation. Cognitive function was assessed by Montreal Cognitive Assessment (MoCA) scale. The total cSVD score was based on the following markers on MRI: lacune; white matter hyperintensities, microbleed, and enlarged perivascular spaces. The association between total cSVD score and MoCA score was tested via Spearman's analysis and a linear regression model.

**Results:** Among the 289 patients, rates for 0–4 cSVD markers respectively ranged from 30.4 to 2.8%. A multiple linear regression model revealed an inverse correlation between the total cSVD score and MoCA score. The association remained significant after adjusting for gender, age, education, levels of medial temporal lobe atrophy, and classical vascular risk factors [β = −0.729, 95% CI (−1.244, −0.213); *P* = 0.006]. When individual markers were individually analyzed after adjusting for the same factors, only microbleed associated with MoCA score [β = −3.007, 95% CI (−4.533, −1.480), *P* < 0.001].

**Conclusions:** A significant association was demonstrated between total cSVD score and cognitive performance in the outpatients with amnestic disorders.

## Introduction

Cerebral vascular disorder is a major cause of various types of dementia, and cerebral small vessel disease (cSVD) is the most common vascular contributor to cognitive dysfunction (1, 2). Signatures (or markers) of cSVD on MRI include enlarged perivascular spaces (EPVS), white matter hyperintensities (WMHs), recent small subcortical infarct, lacune, cerebral microbleed, and brain *atrophy* (3). The cross-sectional and longitudinal investigations have revealed that cognitive dysfunction can be associated with each of these cSVD markers, especially WMHs and lacune or lacunar infarcts (4–8). The effect of these markers may be additive. A proposed scoring system combines all the four MRI markers into a total cSVD score, to represent the total damage burden of cSVD in the brain (9). Several studies have identified an association between the total cSVD score and cognitive decline in the patients with hypertension or lacunar infarction (10, 11), and community-dwelling elders (12, 13). However, the patients with cognitive impairment in memory clinic differ from these cohorts in some aspects, in whom there are fewer cardiovascular risk factors, and more other coexisting neuropathological changes. The present study enrolled the patients from a memory clinic, most of whom suffered from amnestic complaints, and were with dementia, or at high risk of dementia. Within this population, an association between total cSVD burden and cognitive performance was explored.

## Methods

### Subjects

The patients (*n* = 337) with amnestic complaints, who had valid MRI imaging, were retrospectively enrolled for the period from January 1, 2018 to December 31, 2020. All the patients were from the memory clinic of Peking University People's Hospital, Beijing, China.

The patients were excluded who were with cognitive dysfunction secondary to metabolic, nutritional, or infectious factors, such as vitamin B deficiency, alcoholic brain damage, or hypothyroidism; or brain damage due to trauma, central nervous infection, or tumor. In addition, the patients with severe psychiatric disease, delirium or severe psychiatric symptom that would disturb the cognitive assessment or MRI examination; and WMHs caused by demyelinating disease, radiotherapy, or chemotherapy, were excluded. Due to the difficulty of evaluating the cSVD markers on MRI, the patients with large vessel infarction or severe brain hemorrhage were also not included.

All the subjects underwent a clinical evaluation, neurological examination, and cognitive assessment at the first visit. MRI was completed within 7 days after the first visit. The following clinical data were recorded: age, gender, body mass index (BMI), years of education, history of hypertension, diabetes mellitus, previous stroke, coronary heart disease, hyperlipidemia, previous or current smoking, and laboratory tests for serum glucose and lipids.

### Cognitive Assessment

Cognitive function was evaluated using the Chinese version of the Montreal Cognitive Assessment (MoCA) scale, performed by a trained neuropsychologist at the first visit. Cognitive impairment was identified as MoCA < 26 (an additional point was given when the duration of education was <12 years) (14).

### MRI Imaging

The MRI examination was finished within 7 days after the first visit. All the MRI images were acquired in the Radiology Department of Peking University People's Hospital, using a 3.0 T scanner (MR750 and MR750w, General Electric, Waukesha, WI, USA). The following sequences were obtained for all the individuals: T1-weighted, T2-weighted, diffusion-weighted imaging (DWI), fluid attenuated inversion recovery (FLAIR), and susceptibility weighted imaging (SWI). Details about the sequence parameters were shown in Supplementary Table S1 in the supplementary data. The total cSVD score, ranging from 0 to 4, was created with reference to literatures published (9, 13, 15). One point was added for each of the following cSVD markers: lacune, WMH, microbleed, and EPVS.

According to the STRIVE recommendations, lacune was identified as a fluid-filled cavity of diameter 3–15 mm on all the MRI sequences. The location of the lesion was in the territory of a perforating arteriole (3). One point was awarded if there was one or more lacunae. The microbleeds were defined as homogeneous foci (diameter < 10 mm) with low signal intensity on susceptibility weighted imaging. Based on literatures which indicated that deep microbleeds were related to cSVD more specifically, one point was given to the score if there was one or more deep microbleeds (located in the basal ganglia, internal or external capsule, thalamus, or brainstem) (15–17). EPVS was defined as ovoid, round, or linear lesions with a cerebrospinal fluid-like signal, with diameter < 3 mm. One point was given if there were 10 or more EPVS at the basal ganglia level (18). For WMH, one point was given if the periventricular WMH Fazekas score reached 3, or the deep WMH Fazekas score reached 2, according to the Fazekas scale on FLAIR (19).

As a risk factor and imaging feature of cognitive impairment, medial temporal atrophy (MTA) was also assessed on the MRI, on coronal T1 sequence, rated using a validated visual scale from 0 to 4 (20).

The images and clinical information were independently evaluated by the two neurologic radiologists who were blinded to the reading of others. If there was a divergence in scoring, a consultation was conducted to reach an agreement.

### Statistical Analysis

Data analysis was performed with SPSS 19.0 software (IBM, Armonk, NY, USA). The continuous variables are presented as mean ± SD (normally distributed) or median (interquartile range, IQR) (abnormally distributed). Accordingly, analysis variance or the Kruskal-Wallis test was chosen to analyze the differences between the groups. The categorical variables are presented as *n* (%), and the chi-squared (χ^2^) test was used for comparing the differences between the groups. Spearman's correlation analysis was performed to explore the association between total cSVD score and MoCA score. A multiple linear regression model was chosen to investigate whether total cSVD score was an independent risk factor for MoCA score. Demographic factors were adjusted in the model, such as gender, age, education, and classic cardiovascular risk factors. Because MTA is also recognized as a risk factor for cognitive impairment, the MTA levels were adjusted in the regression model.

## Results

**Figure 1:**
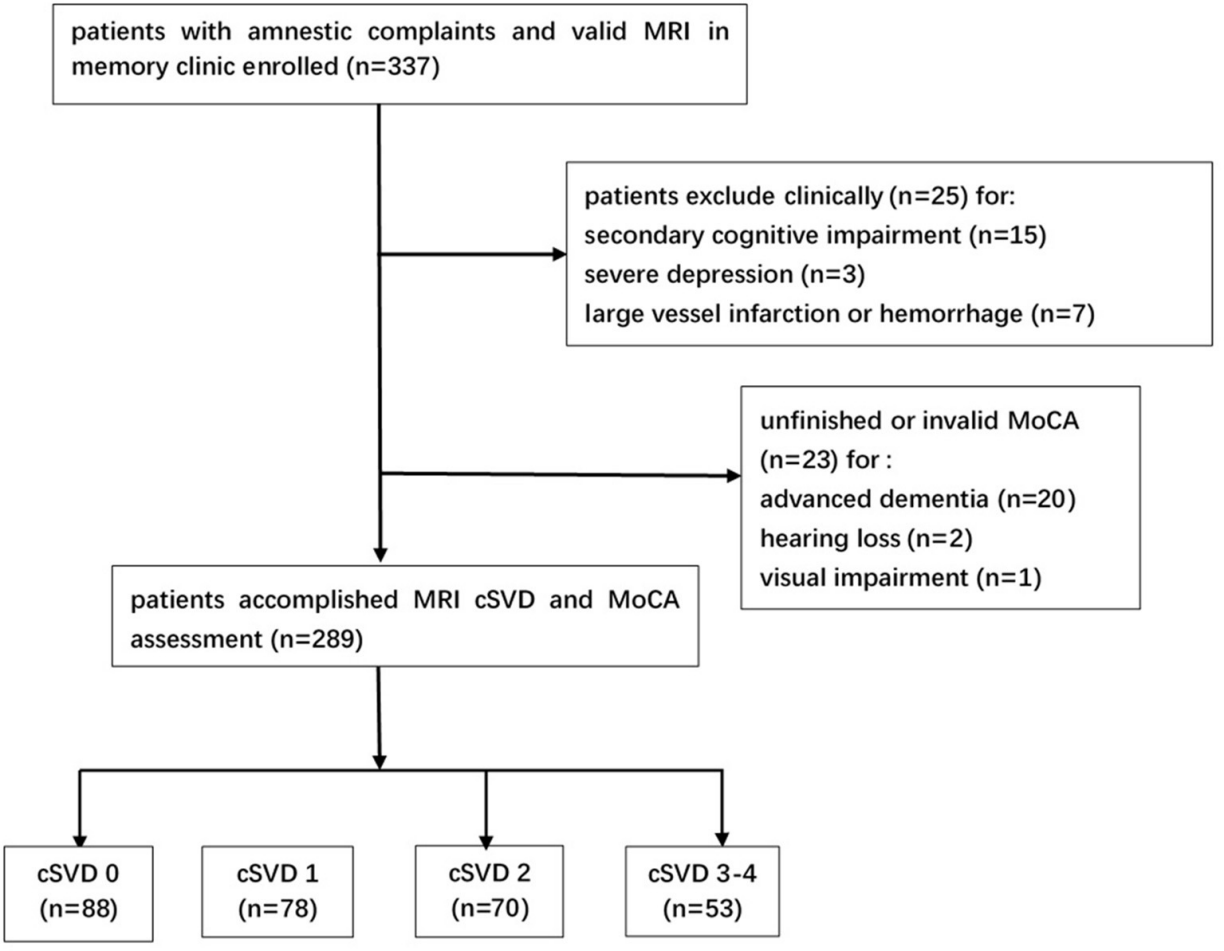
Study flowchart. MRI, magnetic resonance imaging; cSVD, cerebral small vessel disease; MoCA, Montreal Cognitive Assessment.

Among the initial 337 patients enrolled, 25 patients were excluded clinically for brain injury secondary to trauma (two patients), chemotherapy (two patients), hypothyroidism (five patients), vitamin B deficiency (two patients), alcohol (four patients), severe depression (three patients) and large vessel infarctions or severe cerebral hemorrhage (seven patients). Additionally, 23 patients were excluded for unfinished or invalid MoCA assessment. Among them, two patients did not finish MoCA due to the very poor hearing and one patient due to the severe visual impairment (caused by cataract). Twenty patients did not get valid MoCA scale for advanced dementia [16 ones did not finish MoCA, the median MMSE score for these 16 patients was 10.5 (9.0–13.8); and four patients got extremely low and unreliable MoCA scale, the scales for them were 3, 3, 3, and 5] (Figure 1). Finally, 289 subjects were included in this analysis (Table 1). There were no significant differences on demographic characteristics and common vascular risk factors between the subjects included and those excluded except for a higher MMSE score [27.0 (23.0–29.0) vs. 18.0 (10.5–26.0), *P* < 0.001] (Supplementary Table S2 in the supplementary data).

**Table 1:** Baseline characteristics of the subjects with amnestic disorders (*n* = 289)^*^.

**Characteristics**	**All subjects**
Age, y (mean ± SD)	72.4 ± 9.3
Gender, male	119 (41.2)
Education, y	15.0 (9.0–15.0)
Duration before presentation, y	1.8 (1.0–3.0)
BMI, kg/m^2^ (mean ± SD)	25.3 ± 4.7
Hypertension	149 (51.6)
Diabetes	73 (25.3)
Hyperlipidemia	39 (13.5)
Coronary heart disease	40 (13.8)
Previous stroke	24 (8.3)
Smoking	42 (14.5)
Family history	13 (4.5)
MoCA	22.0 (18.0–25.0)
Total cSVD score	1.0 (0–2.0)
EPVS	106 (36.7)
Lacune	150 (51.9)
Microbleed	39 (13.5)
WMH	90 (31.1)

* *Reported as n (%), or median (interquartile range, IQR), unless indicated otherwise*.

*BMI, body mass index; MoCA, Montreal Cognitive Assessment; cSVD, cerebral small vessel disease; EPVS, enlarged perivascular spaces; WMH, white matter hyperintensities*.

**Figure 2:**
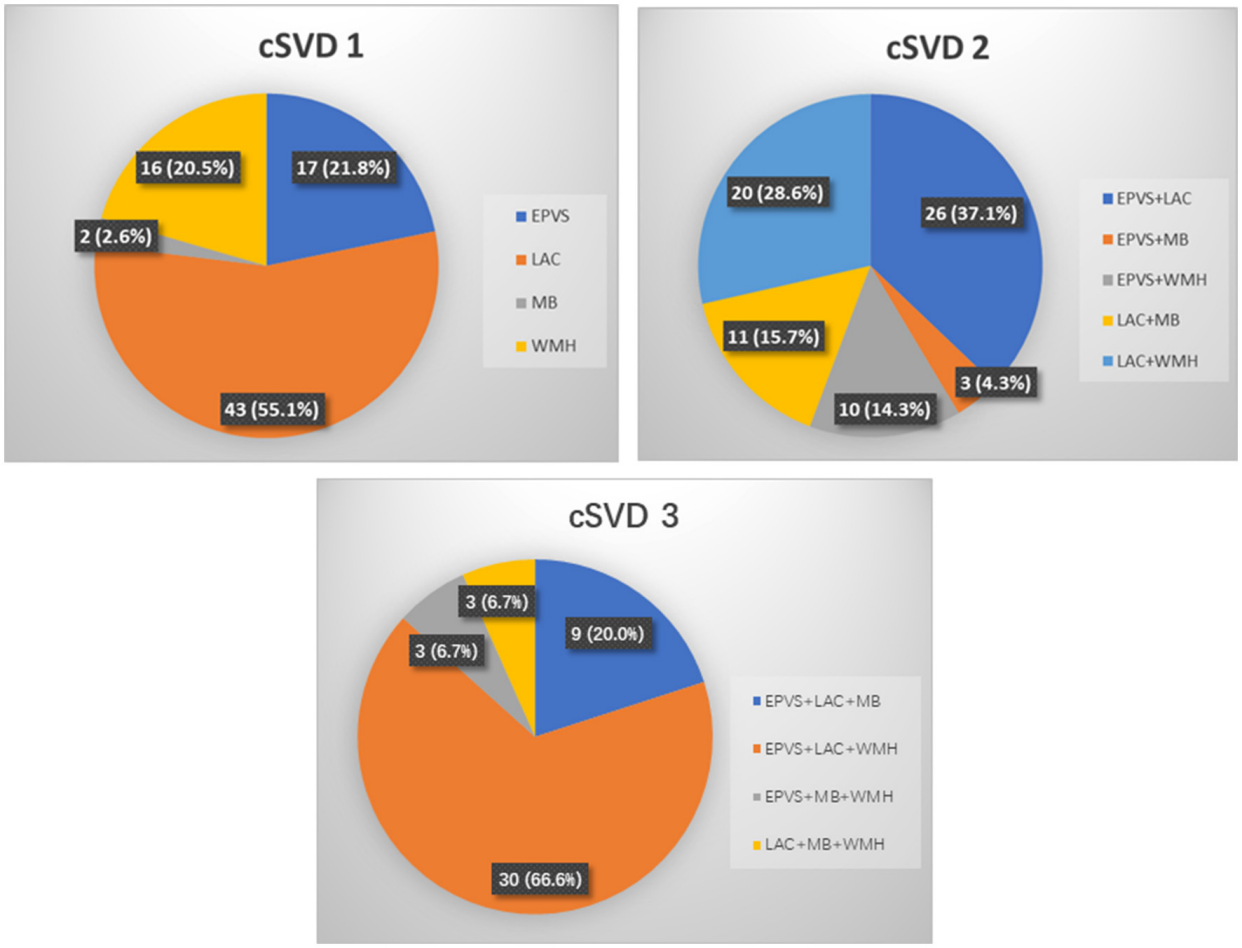
Distribution and combinations of cSVD markers according to the total cSVD score. cSVD, cerebral small vessel disease; EPVS, enlarged perivascular spaces; LAC, lacune; MB, microbleed; WMH, white matter hyperintensities.

Inter-rater reliability for all the cSVD markers was quite acceptable. Cohen κ for periventricular WMH Fazekas score was 0.820 [95% CI (0.767, 0.873), *P* = 0.027], and for deep WMH Fazekas score was 0.815 [95% CI (0.760, 0.870), *P* = 0.028]. Cohen κ for presence of ≥ 10 EPVS was 0.816 [95% CI (0.747, 0.885), *P* < 0.001], presence of ≥ 1 lacune: 0.834 [95% CI (0.771, 0.897), *P* < 0.001] and presence of ≥ 1 microbleed: 0.824 [95% CI (0.732, 0.916), *P* < 0.001]. The number of discordant scores of individual cSVD markers was showed in Supplementary Table S3 of the supplementary data. The MRI scans showed that 30.4% (88/289) of these patients had no markers, and 27.0% (78/289), 24.2% (70/289), 15.6% (45/289), and 2.8% (8/289) had 1, 2, 3, and 4 markers, respectively. The distribution and combination of cSVD markers in different cSVD score was showed in Table 2 and Figure 2. Because there were only eight patients displaying all four markers, the overall population was apportioned to four groups based on the cSVD scores of 0, 1, 2, and ≥ 3 markers.

**Table 2:** Distribution of cSVD markers according to total cSVD score^*^.

	**1 point**	**2 points**	**3 points**
	***n* = 78**	***n* = 70**	***n* = 45**
EPVS	17 (21.8)	39 (55.7)	42 (93.3)
Lacune	43 (55.1)	57 (81.4)	42 (93.3)
Microbleed	2 (2.6)	14 (20)	15 (33.3)
WMH	16 (20.5)	30 (42.9)	36 (80.0)

* *Reported as n (%), unless indicated otherwise*.

*cSVD, cerebral small vessel disease; EPVS, enlarged perivascular spaces; WMH, white matter hyperintensities*.

The patients with higher cSVD scores were significantly older, with higher percentage of previous stroke, higher levels of MTA, and had lower MoCA scores (Table 3). Years of education, the duration of amnestic disorder before visiting, and other clinical factors did not differ among the four cSVD score groups, and there were also no significant differences in the serum glucose or lipid levels.

**Table 3:** The demographic, clinical, and MRI characteristics of patients with amnestic disorders by cSVD score^*^.

	**0 point**	**1 point**	**2 points**	**3–4 points**	***P***
	***n* = 88**	***n* = 78**	***n* = 70**	***n* = 53**	
Age, y (mean ± SD)	67.2 ± 10.7	71.0 ± 8.0	76.0 ± 6.6	78.2 ± 5.5	** < 0.001**
Gender, male	34 (38.6)	31 (39.7)	26 (37.1)	28 (52.8)	0.291
Education, y	15.0 (12.0–15.0)	13.5 (9.0–15.0)	15.0 (9.8–15.0)	15.0 (9.0–15.0)	0.325
Duration before presentation, y	1.0 (0.5–2.5)	2.0 (1.0–3.0)	2.0 (1.0–3.1)	2.0 (1.0–3.0)	0.053
BMI kg/m^2^ (mean ± SD)	25.1 ± 6.4	25.4 ± 3.4	25.1 ± 4.1	25.5 ± 3.4	0.938
Hypertension	38 (43.2)	40 (51.3)	38 (54.3)	33 (62.3)	0.164
Diabetes	26 (29.5)	22 (28.2)	15 (21.4)	10 (18.9)	0.406
Hyperlipidemia	11 (12.5)	15 (19.2)	9 (12.9)	4 (7.5)	0.272
Coronary heart disease	17 (19.3)	10 (12.8)	8 (11.4)	5 (9.4)	0.322
Previous stroke	2 (2.3)	6 (7.7)	7 (10.0)	9 (17.0)	**0.021**
Smoking	14 (15.9)	14 (17.9)	7 (10.0)	7 (13.2)	0.552
Family history	5 (5.7)	6 (7.7)	0 (0)	2 (3.8)	0.139
Fasting glucose, mmol/L	5.5 (5.1–6.4)	5.4 (5.1–5.9)	5.4 (4.9–6.3)	5.6 (5.3–6.3)	0.545
Total cholesterol, mmol/L	4.8 (4.1–5.5)	5.0 (4.1–5.6)	4.2 (3.9–5.2)	4.7 (3.7–5.6)	0.050
Triglycerides, mmol/L	1.4 (1.0–1.9)	1.2 (0.9–1.7)	1.1 (0.9–1.7)	1.3 (0.9–2.2)	0.194
MTA level	1.0 (0–2.0)	1.0 (0.8–2.0)	2.0 (1.0–2.0)	2.0 (1.5–3.0)	** < 0.001**
MoCA	25.0 (21.0–27.0)	23.0 (18.0–25.0)	21.0 (16.0–24.3)	19.0 (15.5–23.0)	** < 0.001**

* *Reported as n (%), or median (IQR), unless indicated otherwise*.

*MRI, magnetic resonance imaging; cSVD, cerebral small vessel disease; BMI, body mass index; MTA, medial temporal atrophy; MoCA, Montreal Cognitive Assessment*.

*Bold values means P < 0.05*.

Spearman's correlation analysis revealed a significant negative association between cSVD score and MoCA score (*r* = −0.377, *P* < 0.001). A linear regression found that high cSVD score was a significant risk factor for low MoCA score [β = −1.667, 95% CI (−2.171, −1.163), *P* < 0.001]. After adjusting for gender, age, education, MTA levels, and classical cardiovascular risk factors, this significance remained [β = −0.729, 95% CI (−1.244, −0.213), *P* = 0.006] (Table 4). When the individual cSVD markers were included in the regression model rather than total cSVD score, the independent risk factors were: lacune, microbleed, and WMH. After adjusting for the same confounding factors mentioned above, only microbleed was a significant independent risk factor for low MoCA score [β = −3.007, 95% CI (−4.533, −1.480), *P* < 0.001].

**Table 4:** Association between cSVD and MoCA scores in the linear regression.

	**Simple linear regression**		**Multiple linear regression**		**Multiple linear regression**	
			**Model 1**		**Model 2**	
	**B (95% CI)**	***P***	**B (95% CI)**	***P***	**B (95% CI)**	***P***
Total cSVD score	−1.667(−2.171, −1.163)	** < 0.001**	−0.809(−1.317, −0.302)	**0.002**	−0.729 (−1.244, −0.213)	**0.006**
EPVS	−0.839 (−2.114, 0.437)	0.197	−0.347 (−1.494, 0.801)	0.552	−0.387 (−1.539, 0.764)	0.509
Lacune	−1.347 (−2.565, −0.128)	**0.030**	−0.533 (−1.624, 0.559)	0.337	−0.360 (−1.459, 0.738)	0.519
Microbleed	−3.994 (−5.724, −2.264)	** < 0.001**	−3.006 (−4.532, −1.480)	** < 0.001**	−3.007 (−4.533, −1.480)	** < 0.001**
WMH	−1.681 (−2.982, −0.380)	**0.011**	−0.336 (−1.516, 0.844)	0.576	−0.148 (−1.339, 1.043)	0.807

*Model 1 was adjusted for age, gender, education, and MTA. Model 2: Model 1+ hypertension, diabetes mellitus, hyperlipidemia, and previous stroke*.

*cSVD, cerebral small vessel disease; MoCA, Montreal Cognitive Assessment; MTA, medial temporal atrophy; EPVS, enlarged perivascular spaces; WMH, white matter hyperintensities; B, nonstandardized coefficients; CI, confidence interval*.

*Bold values means P < 0.05*.

## Discussion

This study investigated the influence of cSVD on cognitive status in a group of patients from a memory clinic. It was found that the cSVD burden, as reflected by the total cSVD score on MRI, was associated with decreased cognitive function, measured by the MoCA scale. Moreover, among the four cSVD markers, only microbleed was a significant independent risk factor for cognitive impairment in this cohort.

There have been a good number of studies of a link between cSVD and cognitive impairment, but most have focused on the influence of individual cSVD markers (5–8), or the combination of two markers (21). However, a total cSVD score has been proposed to reflect the total burden of cSVD on MRI (9, 15). Several studies showed an inverse association between total cSVD score and cognitive performance in the elder patients (12, 13) or in the patients with lacunar stroke or hypertension (10, 22). In contrast to these studies, we recruited a group of patients from a memory clinic whose main complaint was amnestic disorders; only 8.4% had a history of previous stroke. The results were similar to that of study populations with a higher incidence of cerebrovascular disease.

The total cSVD score of the present cohort [1.0 (0–2.0)] suggested a lower burden of cSVD compared with that of another memory clinic [2.0 (0–3.0)] (23), and smaller percentage of lacune, microbleed, and WMH, but the total cSVD score and cognitive dysfunction were similar. Rather than testing the different cognitive domains, we chose the MoCA scale to measure overall cognitive function, which is more practical in daily clinical work and screening. Considering another study about cSVD and cognition that used the MoCA scale in a population of community-dwelling elders (12), the participants in the present study were older (72.4 ± 9.3 cf. 70.1 ± 8.1), but better educated [15.0 (9.0–15.0) years of education cf. 79% primary school only], and the MoCA score was higher (21.2 ± 5.3 cf. 19.2 ± 4.8). Both studies showed a significantly negative association between total cSVD score and MoCA score.

The present research adds to the evidence of a link between cSVD and cognitive impairment, as well as verification for using the total cSVD score to study cSVD and cognition. Only a small portion of our patients had suffered stroke, and most of them had mild-to-moderate cognitive impairment [median MoCA score: 22.0 (18.0–25.0)]. Although the causal relationship between cSVD burden and cognitive impairment could be not identified in retrospective studies, the results indicate that cSVD might play an important role in cognitive dysfunction, even in people without obvious symptom of stroke, with a lower cSVD burden, and in the early stage of cognitive impairment. And the role of cSVD in the development of cognitive impairment need to be clarified in more longitudinal studies.

Several mechanisms have been proposed for cognitive damage caused by cSVD. One explanation is that subcortical damage caused by WMH and lacunae disrupts the integrity of the brain network, which impairs cognitive function that relies on cortical integration (23–25), and subsequently, structural disconnection leads to secondary cortical atrophy (26). Another underlying mechanism is the hypoperfusion and blood brain barrier leakage caused by neuro-glio-vascular damage in cSVD (27). Decreased regional cerebral blood flow was observed in cSVD, and local increase of cerebral blood flow in specific areas in response to neuronal activity was impaired. Hypoperfusion and ischemic damage occurs in the tissues vulnerable to ischemia, such as the hippocampus and the neocortex. On the other hand, dysfunction of the blood brain barrier leads to the leakage of fluids, proteins, and other plasma components into the perivascular tissues and further impairs oxygen and nutrient transport. Moreover, these components might activate inflammation or block amyloid β clearance and then cause Aβ plaque deposit and neuronal damage (28, 29). A third explanation is that cSVD lesions, such as EPVS and WMH, disrupt the interstitial fluid drainage of the glymphatic system, causing impaired clearance of metabolites (β amyloid and other neurotoxic proteins) and abnormal protein elimination (30, 31).

In the present study, when each marker was analyzed individually rather than the total cSVD score, only microbleed was a significant risk factor of lower MoCA performance. Recent studies identified microbleed as a risk factor for cognitive decline (5, 32), and our research provides further evidence of this. Other cSVD markers, especially WMH and lacune, have been significantly associated with cognitive impairment (4, 6–8), but this was not found in the present study. This may be because of the difference in cohorts; our participants showed a higher cSVD score, were much older, and had a remarkably higher incidence of microbleed. This may explain the significant association between microbleed and MoCA score and its dominant contribution in cSVD impact on cognition. In addition, the dichotomization of every marker in this scoring system led to a great loss of information about the severity of the MRI features (13), and thus the relative contribution of the individual markers in the regression is not recognized. Finally, and most importantly, an accumulative effect of individual cSVD features has been implied on global cognitive ability (13), so it is quite reasonable that the addition of several insignificant contributors lead to the final significant impact. It indicates from another side that it is quite necessary to use total cSVD burden instead of individual markers for studying cSVD as a whole.

The strengths of our study include the focus on a cognitively impaired population in a memory clinic, in which a significant association between cSVD burden and cognitive performance was revealed. Few studies of a similar cohort are reported. We chose the total cSVD score on MRI to represent the cSVD burden instead of individual markers, which treated the cSVD more globally and took the accumulative effect of individual markers into consideration. It is a useful method to represent the global cSVD burden in research and practice, even there is some information loss. The MoCA score was chosen for cognitive function. Both the total cSVD score and MoCA score are quite available and reliable in daily clinical work. This study confirms the viability of routinely applying cSVD evaluation in the memory clinic. Furthermore, this study confirms a significant link between microbleed and lowered cognitive performance that echoes the previous studies.

This research may be limited by subjectivity in the visual rating of the MRI, and the dichotomized scoring of each individual marker may overlook information. For improvement, a modified cSVD score was used to account for the severity of the individual cSVD markers, and this improved the identification of patients with vascular cognitive impairment (11, 33). Moreover, a recent study used artificial intelligence to evaluate the global cSVD burden quantitatively (the volumetric data of WMH, lacunes, EPVS, and chronic cortical infarcts) and found it a powerful predictor of long-term cognitive decline (34). The more objective and quantitative evaluation of cSVD burden is necessary for in-depth study on cSVD and it would be used more widely in the future. On the other hand, the dichotomization scale has its advantages, for example, it is relatively simple to use, less time-consuming and need no specific software. It would still be helpful for the primary evaluation of cSVD, especially for screening. In our opinion, the combination of the two methods for cSVD evaluation would be a better choice in clinical practice in the future. Also of note, as we have mentioned, the present study was retrospective cross-sectional research, with data from a single center with limited samples. More longitudinal multicenter studies are needed to investigate the long-term effects of cSVD on cognition.

## Conclusion

This study showed a significant association between total cSVD score and cognitive impairment in a group of patients from a memory clinic. This is further evidence of the value of the total cSVD score for evaluating cSVD. These results warrant further work to explore the role of cSVD in cognition impairment, with larger cohorts, longer follow-up time, and a more accurate assessing system for cSVD burden.

## Data Availability Statement

The raw data supporting the conclusions of this article will be made available by the authors, without undue reservation.

## Ethics Statement

The studies involving human participants were reviewed and approved by the Ethics Committee of Peking University People's Hospital. Written informed consent for participation was not required for this study in accordance with the national legislation and the institutional requirements.

## Author Contributions

YF collected, analyzed and interpreted the patient data, and was a major contributor in writing the manuscript. YX helped to analyze the data and was another major contributor in writing the manuscript. MS made contributions to the acquisition of data. HG revised the manuscript and helped to interpret the data. ZZ designed the present study and provided funding support. All the authors have read and approved the final version of this manuscript.

## Funding

This study was supported by the Shandong Provincial Natural Science Foundation, China (Grants No. 2017G006021).

## Conflict of Interest

The authors declare that the research was conducted in the absence of any commercial or financial relationships that could be construed as a potential conflict of interest.

## Publisher's Note

All claims expressed in this article are solely those of the authors and do not necessarily represent those of their affiliated organizations, or those of the publisher, the editors and the reviewers. Any product that may be evaluated in this article, or claim that may be made by its manufacturer, is not guaranteed or endorsed by the publisher.

## Abbreviations

cSVD: cerebral small vessel disease
MRI: magnetic resonance imaging
MoCA: Montreal Cognitive Assessment
MTA: medial temporal atrophy
WMH: white matter hyperintensities
EPVS: enlarged perivascular spaces.
