# Supplementary material for: Total Cerebral Small Vessel Disease Burden on MRI Correlates With Cognitive Impairment in Outpatients With Amnestic Disorders
Source: Front Neurol. 2021 Dec 2;12:747115. doi: 10.3389/fneur.2021.747115 (PMC8675386; doi:10.3389/fneur.2021.747115)
Supplement: Supplementary file 1 [file Table_1.DOCX]

Supplementary data

Table s-1 MRI Imaging sequence parameters

| MRI sequence | MRI parameters |
| --- | --- |
| TR/TE (ms) T1W | 2390/10.69 |
| TR/TE (ms) T2W | 5200/107.41 |
| TR/TE (ms) FLAIR | 7902/140.45 |
| TR/TE (ms) DWI | 5400/90.30 |
| TR/TE (ms) SWI | 30/7/20 |
| Matrix | 384x286 (T1W)  320x320 (T2W)  288 x320 (FLAIR)  128 x128 (DWI)  448x336 (SWI) |
| Slice thickness (mm) | 5 |
| Inter-slice gap (mm) | 1.5 |

Abbreviations: TR: repetition time; TE: echo time; DWI: diffusion-weighted imaging; FLAIR: fluid attenuated inversion recovery; SWI: susceptibility weighted imaging.

Table s-2 Baseline characteristics of the subjects included and excluded

| Characteristics | Subjects included  n = 289 | Subjects excluded  n = 48 | P |
| --- | --- | --- | --- |
| Age, y (mean ± SD) | 72.4 ± 9.3 | 72.9 ± 10.2 | 0.747 |
| Gender, male | 119 (41.2) | 19 (39.6) | 0.835 |
| Education, y | 15.0 (9.0–15.0) | 12.0 (6.8–12.0) | 0.060 |
| Duration before presentation, y | 1.8 (1.0–3.0) | 2.0 (0.5–3.0) | 0.999 |
| BMI, kg/m^2^ (mean ± SD) | 25.3 ± 4.7 | 24.7 ± 2.9 | 0.454 |
| Hypertension | 149 (51.6) | 18 (37.5) | 0.071 |
| Diabetes | 73 (25.3) | 12 (25.0) | 0.969 |
| Hyperlipidemia | 39 (13.5) | 4 (8.3) | 0.321 |
| Coronary heart disease | 40 (13.8) | 5 (10.4) | 0.518 |
| Previous stroke | 24 (8.3) | 5 (10.4) | 0.629 |
| Smoking | 42 (14.5) | 9 (18.8) | 0.450 |
| Family history | 13 (4.5) | 4 (8.3) | 0.261 |
| MMSE | 27.0 (23.0–29.0) | 18.0 (10.5-26.0) | **< 0.001** |

Reported as *n* (%), or median (IQR), unless indicated otherwise

Abbreviations: BMI: body mass index; MMSE: Mini-Mental State Examination.

Table s-3 number of discordant scores of individual cSVD markers

| cSVD markers | Number of discordant scores n (%) |
| --- | --- |
| EPVS | 25 (8.7) |
| Lacune | 24 (8.3) |
| Microbleed | 13 (4.5) |
| Periventricular WMH | 39 (13.5) |
| Deep WMH | 37 (12.8) |

Abbreviations: cSVD: cerebral small vessel disease; EPVS: enlarged perivascular space; WMH: white matter hyperintensities.
